# Advancements in Public First Responder Programs for Out-of-Hospital Cardiac Arrest: An Updated Literature Review

**DOI:** 10.31083/RCM26140

**Published:** 2025-01-22

**Authors:** Michael Kern, Gerrit Jansen, Bernd Strickmann, Thoralf Kerner

**Affiliations:** ^1^Department of Anesthesiology, Intensive Care Medicine, Emergency Medicine, Pain and Palliative Therapy, Asklepios Klinikum Harburg, 21075 Hamburg, Germany; ^2^Asklepios Campus Hamburg Asklepios Medical School GmbH, 20099 Hamburg, Germany; ^3^University Department of Anesthesiology, Intensive Care Medicine and Emergency Medicine, Johannes Wesling Klinikum Minden, Ruhr University Bochum, 32423 Minden, Germany; ^4^Medical School and University Medical Center East Westphalia-Lippe, University of Bielefeld, 33615 Bielefeld, Germany; ^5^Department of Medical and Emergency Services, Study Institute Westfalen-Lippe, 33602 Bielefeld, Germany; ^6^Bevoelkerungsschutz, District of Guetersloh, 33334 Guetersloh, Germany

**Keywords:** out-of-hospital cardiac arrest, volunteer first responder, AED, smartphone alert, drones, COVID-19

## Abstract

Out-of-hospital cardiac arrest (OHCA) is a leading cause of death worldwide, with a low survival rate of around 7% globally. Key factors for improving survival include witnessed arrest, bystander cardiopulmonary resuscitation (CPR), and early defibrillation. Despite guidelines advocating for the “chain of survival”, bystander CPR and defibrillation rates remain suboptimal. Innovative approaches, such as dispatcher-assisted CPR (DA-CPR) and smartphone-based alerts, have emerged to address these challenges. DA-CPR effectively transforms emergency callers into lay rescuers, and smartphone apps are increasingly being used to alert volunteer first responders to OHCA incidents, enhancing response times and increasing survival rates. Smartphone-based systems offer advantages over traditional text messaging by providing real-time guidance and automated external defibrillator (AED) locations. Studies show improved outcomes with app-based alerts, including higher rates of early CPR, increased survival rates and improved neurological outcomes. Additionally, the potential of unmanned aerial vehicles (drones) to deliver AEDs rapidly to OHCA sites has been demonstrated, particularly in rural areas with extended emergency medical services response times. Despite technological advancements, challenges such as ensuring responder training, effective dispatching, and maintaining responder well-being, particularly during the coronavirus disease 19 (COVID-19) pandemic, remain. During the pandemic, some community first responder programs were suspended or modified due to shortages of personal protective equipment (PPE) and increased risks of infection. However, systems that adapted by using PPE and revising protocols generally maintained responder participation and effectiveness. Moving forward, integrating new technology within robust responder systems and support mechanisms will be essential to improving OHCA outcomes and sustaining effective response networks.

## 1. Introduction 

Out-of-hospital cardiac arrest (OHCA) remains one of the leading causes of death outside hospitals. Worldwide it is estimated that 55 adults per 100,000 person-years suffer from OHCA of a cardiac etiology, 27% with ventricular fibrillation as the initial rhythm [1]. In the U.S., approximately 300,000 people suffer OHCA annually, with a 92% fatality rate [2]. Across Europe, the annual incidence varies from 67 to 170 cases per 100,000 residents. Survival rates following OHCA are generally low, averaging 7% globally and 8% in Europe [1, 3]. Witnessed cardiac arrest, bystander cardiopulmonary resuscitation (CPR), and early defibrillation are crucial factors for improving survival in OHCA, with survival rates declining by 5.5% per minute without intervention [4, 5, 6]. In 2005 the European Resuscitation Council introduced the chain of survival, emphasizing early recognition and call for help, early CPR, and early defibrillation as critical links to optimize outcomes [7, 8, 9]. The American Heart Association has similar guidelines [10].

Nevertheless, even after the introduction of the chain of survival, bystander CPR has still not reached its full potential. International guidelines support various community initiatives to increase bystander CPR rates, but overall, bystander CPR and defibrillation rates remain low, and these efforts often demonstrate limited cost-effectiveness [8, 11]. Bystander CPR rates in Europe vary widely, averaging 58% [12]. Most OHCAs occur at home, reducing the impact of community training [13, 14, 15, 16, 17]. To address this, dispatcher-assisted CPR (DA-CPR) guides callers through CPR, effectively converting callers witnessing OHCA into lay rescuers, therefore significantly improving survival rates [13]. DA-CPR is now recommended by the European Resuscitation Council, the American Heart Association, and the International Liaison Committee on Resuscitation (ILCOR) [8, 18, 19].

However, there are several barriers hindering the transition of callers into lay rescuers. Barriers to effective CPR include the elderly demographic of most OHCA victims (and their spouses) and the emotional and physical limitations of callers [20]. Only 11% of OHCAs are witnessed by emergency services, and with less than 50% of bystanders starting CPR, (trained) community first responders (CFR) are alerted to help fill the gap [21, 22]. Directed by mobile technology and global positioning system (GPS), the goal is to initiate early basic life support (BLS), while professional responders, including professional first responders like fireman or police officers, are still en route [23, 24, 25]. The use of mobile technology in out-of-hospital emergencies, first introduced in 2007, has become crucial in the chain of survival, spreading across many countries [26, 27, 28]. Designed to reduce the therapy-free interval in OHCA, these systems often exclude non-cardiac emergencies and high-risk environments [15, 16, 23, 29, 30].

Early programs used short message service (SMS) to alert lay responders via local emergency dispatch centers, notifying CFR of nearby OHCA incidents [25]. Modern responder systems incorporate mobile apps, which surpass SMS restricted one-way communication by providing automated external defibrillator (AED) locations, enabling on-site audio and video streaming, and directing responders to the closest AED or directly to the victims based on estimated travel times [15, 28]. Fifth-generation (5G) networks will further enhance these systems with high-quality, low-latency communication and precise geolocation [28]. Apps have been shown to get responders to the scene faster and initiate CPR earlier compared to SMS alerts. App-based alerts also resulted in increased survival to hospital discharge in both shockable and non-shockable rhythms [31]. A systematic review of 12 systems in the US, Europe and Asia revealed that nearly 60% of the systems now use app-based alerts, with some even transitioning from text messages to apps. These apps are mostly available on iOS and Android, with limited availability on other platforms [28].

This article aims to provide a comprehensive update on recent developments in (smartphone-based) public first responder alert systems, while also considering future perspectives. Building on the 2020 reviews by Valeriano *et al*. [26] and Scquizzato *et al*. [28], which examined concepts for alerting lay responders to OHCA and summarized the existing evidence, we aim to emphasize recent advancements in this area. To achieve this, the authors performed a PubMed search using keywords such as “first responder app”, “smartphone first responder”, “first responder drones”, and “first responder COVID-19”. Relevant articles were selected if they provided updated information or introduced new insights beyond the previously referenced reviews.

## 2. Outcome Effects 

How does the implementation of smartphone-based alerts for CFRs affect the outcome of OHCA? Scquizzato *et al*. [28] reviewed 28 manuscripts covering 12 first responder systems using apps or text messages. They performed a pooled analysis of 3 studies with 4282 OHCA patients, comparing first responders to standard emergency medical service (EMS). Early CPR before ambulance arrival was higher in the app/text group (63.8% vs. 55%). While return of spontaneous circulation (ROSC) rates were not significantly different (18.9% vs. 12.6%), hospital survival to discharge or survival after 30 days was higher (14.4% vs. 9.4%) in the app/text group than in the standard EMS response group [28]. A study in Seoul showed improved survival and neurological outcomes with a text message alert system [32]. Similarly, the “GoodSAM” system in London and the East Midlands, despite low alert acceptance, improved survival rates [33]. Text message alert systems enhance OHCA care, even in high-survival regions. A study in the Netherlands showed that text message responders reached 15% of OHCA victims earlier, initiated more CPR, and connected more AEDs, thereby improving ROSC rates. Although long-term survival did not significantly improve due to already high baseline rates, alerting responders contributed to optimization of the chain of survival [24].

As urbanization decreases in Sweden beyond the cities, EMS response times exceed 10 minutes in less populated areas. In contrast, volunteer first responder (VFR) response times remain around 4–5 minutes in both low and high density areas, highlighting the potential of VFRs in regions with longer EMS times [23]. As shown by studies in the Netherlands and Denmark, it appears that volunteer responders (VR) hold greater potential in regions with prolonged EMS response times [29, 34]. In Guetersloh, Germany, VFRs arrived before EMS in over 90% of cases, despite median EMS response times of around 7 minutes [35]. Besides shortening arrival times, increased first responder density also reduces defibrillation time in residential areas [36].

## 3. AED Use in Smartphone Alerts 

### 3.1 Utilization of AEDs

The occurrence of ventricular fibrillation and pulseless ventricular tachycardia decreases rapidly in the initial minutes following collapse, as shockable rhythms transition into asystole [23]. This decline in shockable rhythms diminishes the likelihood of surviving a cardiac arrest while awaiting early defibrillation treatment—a crucial factor strongly associated with increased survival rates [37, 38]. Early defibrillation is critical for survival in patients with ventricular fibrillation or pulseless ventricular tachycardia, but the use of public access defibrillators in OHCA remains low, especially in residential areas [3, 39]. Although AEDs are available in public spaces, bystanders use them in less than 2% of OHCA cases in the US [40]. Public AED networks encounter various operational challenges: most people don’t know the nearest location, first responders need to pick up the AED by foot, and AEDs often have low accessibility, especially in residential areas—as most defibrillators are kept in enclosed spaces such as offices. Potential obstacles to guide bystanders to the nearest AED include roads, staircases, locked doors, and the lack of regulated or standardized AED signage [40]. These issues increase access time and divert attention from performing CPR [41]. Several programs, including ensuring public access to defibrillators and equipping non-medical emergency personnel with AEDs, aim to increase early defibrillation rates [4, 42, 43, 44]. In addition to the concept of engaging individuals from various professions like taxi drivers, food-delivery workers, mail carriers, and police officers to retrieve and transport AEDs to the scene, another option is to equip certified lay responders with compact AEDs that can be easily transported on bicycles, motorcycles, or in private cars [15, 28]. Using vehicles to reach OHCA scenes and issuing “personal” AEDs to trained lay responders could reduce defibrillation time by eliminating the need for AED pickup [15].

Most OHCA cases occur at home among elderly males, with lower survival rates compared to public areas due to less bystander CPR, older patients with more comorbidities, and fewer shockable rhythms [45, 46]. AED use by bystanders seldom contributes to defibrillation in residential areas, partly due to the low availability of AEDs in these areas [34, 45]. Most devices are located in public areas such as offices, schools, and shopping malls, which results in limited 24/7 accessibility and hinders early defibrillation [47]. By dispatching laypersons to retrieve public access defibrillators, several smartphone-based alert systems aim to enhance early defibrillation rates, particularly in residential areas. Some systems dispatch the initial citizen-responders primarily to the nearest accessible AED and subsequently to the patient’s address [16]. A second dispatch method prioritizes the initiation of CPR by directing the first responders straight to the scene of the OHCA, while instructing the following responders to retrieve the nearest available public access defibrillator [25]. A third strategy is instructing the nearest first responder (FR) to collect an AED, sending the remaining FR directly to the patient [28]. A 2019 review found that 18 out of 25 systems used AED location databases [26]. In simulation trials verbal directions to AEDs saved time compared to geo-localization via mobile apps [40, 48].

The American Heart Association recommends placing AEDs within a 1–1.5 minute “brisk walk” to OHCA locations (translating to a straight line distance of 100 m) [47]. Studies in London, the East Midlands, and Switzerland indicate that sending responders directly to perform CPR might be more effective than retrieving AEDs first, having to walk 400 m or more to the nearest AED, resulting in a median delay of 78 seconds in reaching the victim [15, 49]. This delay corresponds to a reduction in the chances of survival ranging from 10% to 15% [15]. Berglund *et al*. [23] demonstrated that in Sweden, EMS, firefighters, and AED-equipped responders rallied to the scene, arriving almost within a minute. Only sending volunteers directly to the scene to initiate CPR resulted in a significant time advantage for volunteers [23]. Therefore, directing all laypersons to start CPR, while waiting for professionals to bring the AED, may be the better option.

Conversely, patients with a non-shockable rhythm upon the arrival of EMS might still have had a shockable rhythm, (as an initially shockable rhythm will deteriorate into asystole when treatment is not initiated promptly [23]), if lay responders would have arrived before the professionals [45]. The benefit of early defibrillation remains futile if lay responders fail to bring an AED to the scene. Alerting volunteers and giving them directions to the nearest defibrillator enhances the use of AEDs and shortens the time from emergency call to defibrillation compared to EMS alert alone [25]. Volunteer first responders in Denmark, directed to AEDs via GPS tracking, arrived significantly faster than EMS. The median response time for VFRs was 4 minutes and 46 seconds and it took VFRs 6 minutes and 21 seconds to arrive with an AED. In contrast, EMS units required over 10 minutes to reach the scene [46]. In the Netherlands time to first defibrillation decreased, if at least one VFR in residential areas was directed through an AED location, reducing the likelihood of EMS being the first to administer defibrillation (decreasing from 63% to 44%). This highlights the potential for early AED use by first responders [36]. In residential areas, 16% of initial defibrillations were carried out by text message (TM) responders in North-Holland, underlying the benefit of sending TM to obtain AEDs in residential areas. As a result, survival rates in residential areas were nearing those observed in public [34]. Alerting volunteers and directing them to the nearest defibrillator seems to enhance AED use and shortens the time from the emergency call to defibrillation compared to EMS alert alone.

### 3.2 Using New Technologies – Years of the Drones?

With technology evolving, Unmanned Aerial Vehicles (UAVs), or drones, are now accessible for non-military applications. Drones with the ability to carry AEDs and bring them to OHCA sites present a potential application of this new technology in the chain of survival. This innovation spares volunteer responders to run “the extra” mile to obtain an AED, while also providing higher accessibility of AEDs. In a simulated study in Stockholm and the surrounding rural area, UAVs could arrive before EMS in 32% of OHCA calls in the urban setting (saving 1.5 minutes) and in 93% of rural cases (saving 19 minutes), demonstrating that the most significant benefit of drones can be found in rural areas with longer EMS response times [50]. In a U.S. county, 98% of the suburban population could receive an AED via drone in under 10 minutes, five times faster than ground EMS units would have been able to provide defibrillation, as shown by Ryan *et al*. in 2021 [51]. UAVs can take off within 10 seconds after dispatch, offering advantages like direct travel paths and independence from traffic patterns as well as a reduced susceptibility to staffing shortages. Drones can commence their operations after dispatch in as little as 10 seconds. Land-based EMS response time is known to be up to 90 seconds after dispatch, while helicopters usually take up to 5 minutes before they are airborne [50, 52]. In a simulation study in rural Germany, UAVs equipped with AEDs landed safely at simulated OHCA sites, with bystanders and VFRs able to retrieve the AEDs without safety concerns. Interviews revealed strong support for this system, with 95% of bystanders and 100% of VFRs in favor of AED delivery [53]. Combining drone AED delivery with VFR dispatch ensures continuous high-quality CPR and shortens defibrillation time.

**Fig. 1.**
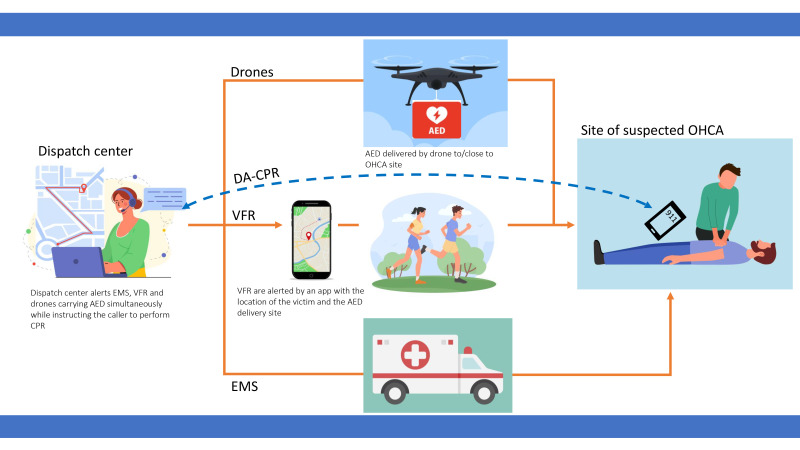
**Incorporating automated external defibrillator (AED) delivery by drone into the chain of survival**. After a 911 call for a suspected out-of-hospital cardiac arrest (OHCA), the dispatch center alerts emergency medical service (EMS) and volunteer first responder (VFR) while providing dispatcher-assisted cardiopulmonary resuscitation (DA-CPR) to the caller. Simultaneously, a drone carrying an AED is dispatched to the OHCA site, delivering the AED for use by VFR. The location of the victim and the AED delivery site are displayed on the smartphone app used by the VFR.

Successfully integrating drones in real life emergencies as part of the chain of survival within dispatch centers, EMS, and first and volunteer responders, is one of the key challenges when using drones for AED delivery. Amidst rising ambulance arrival times in Sweden, Schierbeck *et al*. [54] assessed the practicality of delivering AEDs using drones in real-life OHCA incidents. Over a four-month period, drones equipped with AEDs were dispatched to suspected OHCA incidents, successfully delivering AEDs in 92% of cases with a median delivery time of short over nine minutes from the emergency call. Drones outpaced ambulances in 64% of cases, saving an average of 1 minute and 52 seconds, though no AEDs were attached to victims before ambulance arrival (in this study no (volunteer) first responders were dispatched simultaneously for OHCA calls). The authors anticipate future arrival times to be under 7 minutes [54]. Reducing call-to-defibrillation time to under 8 minutes can save lives, as early AED use prior to EMS arrival can double survival rates based on data from the Swedish CPR register [55]. Integrating AED drone delivery locations into smartphone applications for lay responders could further encourage prompt and widespread AED use, strengthening the chain of survival (Fig. 1).

Incorporating drone delivery of AEDs into the chain of survival, however, necessitates addressing several technical prerequisites: high-resolution cameras, stable mobile connections, and secure, mature software for automatic flight [53]. Drone flights also require prior airspace clearance, which can add to deployment time and reduce time benefits [53, 54]. Other factors like no-fly zones, high buildings, long flight distances, severe weather, maintenance issues, or loss of connection can also impede drone flights [54, 56]. In real life testing and simulations, the majority of bystanders and lay responders in all studies felt comfortable interacting with drones and neither safety issues nor adverse events occurred during any flights [50, 53, 56, 57, 58]. In addition to technical prerequisites, there are also economic considerations for drone networks. A Swedish retrospective observational registry-based study (2010–2018) identified a high-incidence OHCA regions and quantified the number of AED-equipped drones needed to deliver an AED within eight minutes. To achieve a 50% delivery rate within eight minutes, 21 drones were required, while an 80% delivery rate necessitated 366 drones, saving a median of over 3 minutes compared to EMS arrival. Covering all areas, especially those with a low OHCA incidence, would require an exponential increase in drones, with more than a thousand needed to achieve 100% coverage [59]. In rural Germany, a UAV-based AED network would prove more cost-efficient than stationary AED networks, with a four-minute flight radius covering 75% of previous OHCAs hot spots [41].

## 4. Designing Volunteer First Responder Systems

### 4.1 Dispatching

Deployment of VFRs should be done by dispatch centers, as they offer a better integration into the EMS system and are also able to evaluate each mission in terms of safety. Systems working independently from dispatch centers cannot provide specific risk assessment of each mission. A 2021 consensus paper emphasized the need for communication between VFRs and dispatch centers during missions [22]. The American Heart Association recommends dispatching the first unit within 60 seconds of an OHCA call [18]. When VFRs were dispatched more than a minute later than EMS units, as observed in Stockholm and Gothenburg, they lost their time advantage, leading to similar total response times: 10.4 minutes for EMS, 9.6 minutes for AED-responders, and 8.2 minutes for VFRs. A simultaneous dispatch of the VFRs with the first EMS unit would have led to a 1 minute gain in the total response time, allowing VFRs to arrive and start BLS earlier [23]. Stroop *et al*. [35] showed that concurrent alerts of CPR-trained VFRs and EMS improved response times, hospital discharge rates, and neurological outcomes in parts of Germany. Patients treated by first responders had a survival rate of 11% with good neurological outcomes, defined as a Cerebral Performance Categories (CPC) score of 1 or 2, compared to just 5% in the group treated solely by EMS [35]. For supplementary resources like firefighters and volunteer responders, dispatch should occur concurrently with or even before the dispatch of EMS units.

### 4.2 Qualification of First Responders

Required medical qualifications to participate as a volunteer responder vary from system to system. Many systems register laypersons, who are only qualified as BLS-providers [60], some requiring yearly mandatory training to renew certification [46]. In Copenhagen, first responders are required to be 18 years or older, and while CPR and/or AED training is highly recommended, it is not mandatory for registration [29]. In Amsterdam, text message responders are non-medical persons who have completed a course in CPR and AED use. A university in Switzerland specifically enlisted medical and dental students as first responders (after a short e-learning course and training by a certified BLS-instructor) [61]. In the UK, registering with the “GoodSAM” app requires a minimum of an up-to-date CPR training, whereas in Australia and New Zealand, the same app does not have this requirement [62, 63]. According to a ministerial directive, smartphone alerting systems in Germany require a qualification as a nurse, physician, paramedic, emergency medical technician, or being a medical student [60]. There is some evidence that OHCA survival rates are doubled if bystander CRP is performed by medical staff rather than laypersons [22]. CPR initiated by volunteers alerted through a mobile app, primarily with a medical background such as nurses, physicians, firefighters or policemen, led to an enhanced neurological outcome compared to CPR initiated by EMS in a region in Germany [35].

### 4.3 Recruitment of and Impact of Calls on Lay Responders

To establish an efficient VFR system, several factors must be considered beyond alerting times, drone delivery, or AED availability. Key elements include responder availability and density. Stieglis *et al*. [36] found that a density of more than 10 responders per km^2^ increased early defibrillation. However, having enough registered responders does not guarantee that all alerts are covered, with acceptance rates varying between 30 to 50% [28, 29, 35]. The efficiency of these systems is determined by the prevailing culture of action and education in resuscitation techniques within the population of each respective country [28]. For instance, the “GoodSAM” app showed a 38% acceptance rate in Australia and New Zealand, compared to only 16% in London and 15% in the East Midlands [33, 62]. Denmark’s Langeland island demonstrated a high response rate of 96%, with VFRs arriving before EMS in 85% of alerts, due to rigorous training and annual renewal of certification [46]. These findings suggest that varying levels of education and training may influence commitment to VFR. This hypothesis is reinforced by the findings of Stroop *et al*. [35], indicating a strong commitment among FRs with medical or professional backgrounds, leading to an acceptance rate above the average (almost half of the triggered alarms). In Copenhagen, only 8% of alarms were not accepted, with 98% of responders having received CPR training prior to being alerted [29]. To address obstacles to AED use by VFRs, interviews with London responders revealed a preference for initiating CPR over retrieving AEDs. However, no participant felt unable to perform CPR or use an AED. The authors suggest minimizing obstacles to AED retrieval, emphasizing AED locations, ensuring access to locked cabinets, and displaying route distances and times to the nearest AED and patient [63]. Prior CPR/AED training not only motivates participation but also increases the willingness to use AEDs [39, 62]. Responders with CPR experience were more likely to provide patient care, especially those with medical backgrounds [62].

Apart from experience and training, variations in geographical locations and the level of urbanization can lead to divergent response rates. In rural regions characterized by lower AED density and prolonged EMS response times, a heightened sense of commitment among citizens serves as a motivating factor for their participation in VFR networks, as demonstrated on the Island Langeland, Denmark. In this rural community, at least one VFR arrived at the emergency site in 96% of the calls, as shown by Sarkisian *et al*. [46]. This is in contrast to the much more densely populated areas of London, where the acceptance rate is below 20% [33].

A responder’s willingness to respond to an alarm is also affected by the distance to the incident and the perception of whether they might be the first to arrive at the scene [63]. A common complaint among volunteer first responders is the prior experience of ambulance personnel arriving before them [23, 63]. Some systems calculate the estimated arrival of VFRs on the scene and automatically exclude those responders who are expected to arrive after the ambulance, while others only dispatch responders within a certain time reach [15, 64].

## 5. Well-Being of Responders and COVID-19’s Disruption or Keeping Systems Running

### 5.1 Burden on Responders

Enlisting and training VFRs is just one aspect of a working VFR system. Another critical component is ensuring the safety of responders during and after missions. Professional first responders are at high risk for mental disorders such as post-traumatic stress disorder, depression, and problematic alcohol use due to repeated exposure to stress and trauma. Preventive care and supportive systems are being implemented to assure the mental health of all first responders [65, 66]. Andelius *et al*. [29] examined self-reported physical injuries and the psychological impact among activated citizen responders in the capital region of Denmark for all VFR alarms over the course of a year. Participants had the option to report physical injuries and/or the extent of psychological impact. Those who indicated as having been severely affected by the alarm were contacted and offered debriefing by healthcare professionals. Out of 1621 responders 22 (1.4%) reported as having been severely impacted by the alarm. 3 responders received a professional follow-up. The authors also reported 1 physical injury requiring hospital admission (a lower extremity fracture while running to the OHCA location) and three minor injuries. More than 99% of the participants expressed a desire to continue their involvement after being dispatched [29]. In Australia and New Zealand, only 0.6% of all “GoodSAM” responders contacted by phone or email two weeks after an alarm screened positive for probable post-traumatic stress disorder. None of these cases were associated with the smartphone alert. The majority of responders found debriefing beneficial, implying that a formal debrief by telephone would contribute to the well-being of app responders [62]. Although citizen responders seem to be a resilient population and CFR networks appear to be safe, debriefing programs should be available to offer support if needed [29].

### 5.2 Impact of the Coronavirus Disease 19 (COVID-19) Pandemic

The outbreak of the novel severe acute respiratory syndrome coronavirus type 2 causing COVID-19 was declared a pandemic by the World Health Organization in March 2020. In a very short time, healthcare systems and society faced severe challenges. The main objectives were to prevent transmission and slow the rate of infections [67]. CPR, particularly chest compressions, was considered aerosol-generating, leading to adjustments in OHCA management, such as adopting compression-only CPR and covering the patient’s mouth during resuscitation. Professional healthcare workers were advised to wear airborne precaution personal protective equipment (PPE) [68]. Especially in the initial stages of the pandemic, the significant demand for PPE resulted in shortages in certain regions. Supplying equipment to all CFRs was often impractical, given that responders are frequently laypersons, and some programs encompassed thousands of responders [17, 69]. Consequently, some CFR programs were temporarily suspended due to the increased infection risk and the essential role of some first responders in the healthcare system [60, 69, 70]. Other systems remained active but with imposed restrictions. The most common restriction was not to perform rescue breaths and provide compression-only CPR [69]. Approximately 50% of the volunteer systems in Europe, as identified in a cross-sectional survey study, temporarily halted their citizen responder programs (9 out of 18). In systems that continued to operate, responders were provided with PPE [69]. A similar handling of the pandemic could be seen in professional first responder systems across Europe [71]. In the United States, the “PulsePoint” first responder app also remained in operation [72].

The pandemic disrupted the chain of survival, with decreased rates of bystander CPR and AED use, leading to poorer patient outcomes [73, 74, 75]. Communities and regions severely affected by COVID-19 showed an increased incidence of OHCA [75]. Since halting first responder systems created a major disruption in the chain of survival, a COVID-safe restart strategy was devised in Freiburg, Germany. This plan involved supplying CFRs with PPE and conducting a survey to assess their willingness to respond. The survey indicated that a greater number of first responders were willing to respond to calls after being provided with PPE, and even more so after receiving a vaccination. Conversely, without PPE, willingness to respond dropped dramatically [60]. In Australia, despite partial shutdowns of the “GoodSAM” system, most responders indicated they would have responded to alerts during the shutdown, even without PPE. Ball *et al*. [70] observed that paramedics were less likely to respond as CFRs, presumably due to psychological distress and increased exposure to COVID-19 in their work environment.

In Denmark, the CFR system remained active during the lockdown, with no reduction in first responder interventions for OHCA. There was even a slight trend towards increased acceptance rates. The only noticeable change was a shift towards compression-only CPR, as recommended by the citizen responder program at the beginning of the lockdown. Despite government-mandated closure of offices, bars, restaurants, and other publicly accessible places, the availability of AEDs and the distance traveled by CFRs to scenes remained unchanged. Citizen responders reported lower psychological impact during the lockdown, possibly due to the lower incidence of COVID-19 in Denmark and the younger median age of responders [17]. The “Mobile Retter” app in Guetersloh, Germany, which includes volunteer CFRs with medical qualifications and/or certified experience in prehospital emergency care, remained active with only 10% of participants pausing their involvement during the pandemic. During the COVID-19 pandemic and lockdowns, CFRs were alerted less frequently, but their response rates were significantly higher compared to pre-COVID-19. Initiation of CPR by CFRs increased the odds of survival until hospital discharge. While professional paramedics contracted COVID-19 in up to 10% of cases following prehospital CPR, first responders using PPE in this system reported no infections during the pandemic [64].

## 6. Conclusions

OHCA remains one of the leading causes of death outside medical facilities. Enhancing survival rates in OHCA relies significantly on bystander CPR and prompt defibrillation. However, due to the majority of OHCAs taking place at home, the utilization of AEDs remains limited. Empowering volunteer laypersons as first responders through mobile applications or text messages alerts strengthens the chain of survival. The deployment of community first responders is associated with reduced response times for CPR and defibrillation, thereby improving outcomes for OHCA victims. Challenges in implementing lay responder systems include training, efficient dispatching, responder localization and AED retrieval. The objectives of increased bystander CPR and shortened time to defibrillation should be individually assessed for each system. Innovative solutions like unmanned aerial vehicles, commonly known as drones, could play a crucial role in maximizing the potential of volunteer responder systems. Providing personal protective equipment was crucial for sustaining a citizen response system during the COVID-19 pandemic, and debriefing programs should be implemented for the well-being of CFRs following calls.

## Abbreviations

AED, Automated external defibrillator; BLS, Basic life support; CFR, Community first responder; COVID-19, Coronavirus disease 19; CPR, Cardiopulmonary resuscitation; DA-CPR, Dispatcher-assisted cardiopulmonary resuscitation; EMS, Emergency Medical Service; OHCA, Out-of-hospital cardiac arrest; PPE, Personal protective equipment; ROSC, Return of spontaneous circulation; SMS, Short message service; TM, Text message; UAV, Unmanned aerial vehicle; VFR, Volunteer first responder.
